# Alterations of White Matter Structural Brain Network in Children With Sensorineural Hearing Loss: A Graph Theory Analysis for Auditory Sensitivity Period

**DOI:** 10.1155/np/4263849

**Published:** 2026-03-13

**Authors:** Jiayan Zhuang, Jin Wang, Gengbiao Zhang, Hongyi Zheng, Lingmei Kong, Lexing Huang, Juyue Hong, Wenbin Zheng

**Affiliations:** ^1^ Department of Medical Imaging, The Second Affiliated Hospital of Shantou University Medical College, Shantou, 515041, Guangdong Province, China, st120.cn

**Keywords:** auditory sensitive period, brain networks, diffusion tensor imaging, graph theory analysis, sensorineural hearing loss

## Abstract

**Objective:**

The central auditory system has greater plasticity when children are 2–4 years old, called the auditory sensitive period, during which cochlear implantation (CI) can give a good prognosis to children with congenital sensorineural hearing loss (CSNHL), but some will have a poor prognosis. This experiment aims to construct a structural brain network by using diffusion tensor imaging (DTI) and analyze alterations of the topological properties of structural brain networks by graph theory method, provide an imaging basis for the pathophysiological mechanism of white matter network changes with age in SNHL children.

**Materials and Methods:**

A total of 109 children with SNHL and 61 normal control groups were included. According to the auditory sensitivity period, children were divided into a group within the auditory sensitivity period (group A, 12–47 months) and a group beyond the auditory sensitivity period (group B, 48–120 months). DTI data were collected to construct the structural brain network for graph theory analysis, identify the network changes of the topological properties of the SNHL, as well as the differences before and after the auditory sensitivity period.

**Results:**

Compared with the control group, both groups of SNHL had network topological changes. Group A showed a decrease in nodal parameters in higher‐order cognitive‐related brain regions but no difference in global topology parameters and network connection strength. However, group B showed a decrease in the nodal parameters of multiple contact cortical brain regions and the global efficiency (Eglob) of structural networks. Besides, subnetwork analysis showed weakened connection strength between key brain regions related to higher‐order cognition.

**Conclusion:**

The structural brain network of SNHL children before the auditory sensitive period has not undergone extensive changes, and once the auditory sensitive period of development is exceeded, the formation of deaf brain features will be more obvious, with greater impact on higher‐order cognitive regions. Before the auditory sensitivity period, hearing stimulation should be introduced to SNHL children as soon as possible to reduce extensive damage to the white matter network.

## 1. Introduction

Sensorineural hearing loss (SNHL) refers to hearing impairment or loss caused by damage to the inner ear or dysfunction of the sensory receptors. According to statistics from the World Health Organization (WHO), more than 900 million people (1 in 10) will have SNHL disability, which is the most common cause of hearing loss among people with hearing impairment [1]. With the feature of early onset, congenital SNHL (CSNHL) not only has a serious impact on speech and language, cognitive, and emotional development of children but also poses great burdens on individuals, their families, as well as the whole society [2]. Cochlear implantation (CI) should be considered among children with moderate to severe hearing loss. The combination of computed tomography (CT) and magnetic resonance imaging (MRI) remains a common method for screening SNHL and evaluation before CI, which can assess morphological abnormalities in SNHL patients [3]. However, a growing body of evidence has indicated significantly lower average levels of long‐term education and occupation after CI [4]. This SNHL may affect not only the auditory and language systems but also a variety of brain systems and higher functions, including visual system [3], motor balance [5], cognitive status, and working memory. Also, there are individual differences in the degree of recovery after CI [2]. Thus, it can be seen that SNHL may have a larger range of brain network changes beyond the morphological level, whereas the understanding of whole‐brain‐level network changes and the specific mechanism of its effect in SNHL patients has not been studied in depth. Structural changes and remodeling of brain function in children with CSNHL still require further studies.

Brain development is a long process. Early disruptive events may lead to lifelong impacts, such as neuropathological diseases in childhood and adolescence [6]. The developing brain presents remarkable plasticity and is capable of reconstructing synaptic connections based on changing experience. The basic layout of the brain is first established by genetic programs and intrinsic activity, followed by extrinsic environment actively promoting development, and this experience‐dependent neuronal circuit sculpture occurs at different time windows, with the sensitive period being the most obvious [7]. Substantial studies have identified the existence of an auditory sensitive period within the first 2–4 years of SNHL pediatric patients [8–10], during which the structural and functional development of the brain shows explosive growth and the input of stimuli (e.g., hearing and vision) can have a huge impact. In the auditory sensitive period, the central auditory system has the greatest plasticity, and CI can obtain a better clinical prognosis and reduce the occurrence of serious complications such as mental retardation and speech impairment [9, 10]. This may be because myelination and synaptogenesis occur more frequently during the rapid advancement of white matter structure development in early life, providing a material basis for brain plasticity [8, 11, 12]. Previous studies on SNHL children have reached similar conclusions [13–15], that is, compared with healthy children, there appear to be significant developmental delays in the brain in those older than 3–4 years, while those younger than 3–4 years are relatively normal. Some studies on deaf children have demonstrated the negative correlation between age and clinical indicators such as categories of auditory performance (CAP) score [13], as well as experimental measurement data including fractional anisotropy (FA) value and latency of auditory trigger potentials in electroencephalography (EEG) [16]. Some scholars have proposed that white matter fiber myelination and axonal structure in SNHL children may be affected by hearing deprivation, while white matter networks are composed of myelin axons and associated cells that support the transmission and coordination of neural signals [17]. MRI‐based diffusion tensor imaging (DTI) can quantitatively display white matter microstructure in SNHL children, and FA values from DTI measurements correlate well with hearing impairment [18]. Research on SNHL children through DTI analysis has reported changes in both auditory regions (e.g., auditory radiation and Heschl’s gyrus, etc.) and nonauditory regions (e.g., superior longitudinal fasciculus and corticospinal tract, etc.) [12, 13, 16, 19, 20]. With the research progress of brain networks, the use of DTI to reconstruct brain nerve fiber tracts has been carried out. By modeling the corresponding fiber tracts as white matter brain networks [21–23] and using graph theoretical methods for analyzing complex networks, researchers can assess the biological characteristics such as white matter connectivity, topological properties, and information transmission efficiency in brain networks, which has shown good clinical value in a variety of diseases including acute carbon monoxide poisoning [24], autism [25], migraine [26], Parkinson’s disease [27, 28], and traumatic brain injury [29]. However, there are still few applications of DTI combined with complex network graph theory analysis in the study of white matter networks among children with SNHL.

Therefore, we conducted a subgroup analysis to investigate the auditory sensitivity period in children with SNHL. Based on previous findings that synaptic density peaks between ages 2 and 4 [8, 12], we selected 4 years of age as the cutoff to best preserve the integrity of this critical period. This study examines brain network remodeling in SNHL children, emphasizing early hearing stimulation for tailored clinical treatments.

## 2. Materials and Methods

### 2.1. Study Population

The study was approved by the Ethics Committee of the Second Affiliated Hospital of Shantou University Medical College (Number 2022‐11). A total of 159 children were recruited from Oct 2012 to Apr 2025, and divided into SNHL and normal hearing groups. The inclusion criteria were as follows [13, 14, 20, 30]: (1) Auditory brainstem response (ABR) results were greater than 70 dB (i.e., all pediatric patients presented with congenital, severe‐to‐profound SNHL and were all candidates for CI); (2) No inner ear, auditory nerve, or central pathway abnormalities on pre‐op MRI/HRCT; (3) No prior head issues or CNS diseases. After excluding 14 patients for image quality, head movement, or intracranial conditions, 95 SNHL and 61 control children were included.

Subjects received 50–60 mg/kg of 10% chloral hydrate orally before scanning. To minimize scanner noise exposure, all subjects were fitted with dual hearing protection during the MRI scan, specifically insert earplugs (EarSoft FX, 31 dB attenuation) and over‐ear headphones (MR Confon, 20 dB attenuation), which resulted in a total noise reduction of ~50 dB.

### 2.2. Grouping

According to the auditory sensitivity period, the children were divided into a group within the auditory sensitivity period (group A, 12–47 months) and a group beyond the auditory sensitivity period (group B, 48–120 months). Group A included 61 children with SNHL (29 males and 32 females, with a mean age of 27.89 ± 9.18 months) and 30 children with normal hearing (16 males and 14 females, with a mean age of 28.24 ± 10.87 months). Group B included 34 children with SNHL (19 males and 15 females, with a mean age of 78.59 ± 34.45 months) and 31 children with normal hearing (12 males and 19 females, with a mean age of 86.42 ± 29.15 months). No statistically significant differences in gender (chi‐square test) or age (Mann–Whitney *U* test) were observed in either the Group A or Group B SNHL subgroups when compared with their respective normal hearing groups (Table 1).

**Table 1:** Comparison of demographics between SNHL group and normal hearing group.

Group	*n*	Age (months) (mean ± SD)	Sex (M/F)	Age comparison (*p-Value*)	Sex comparison (*p-Value*)
Group A					
SNHL	61	27.89 ± 9.18	29/32	0.880^a^	0.603^b^
Normal hearing	30	28.24 ± 10.87	16/14		
Group B					
SNHL	34	78.59 ± 34.45	19/15	0.095^c^	0.166^b^
Normal hearing	31	86.42 ± 29.15	12/19		

*Note*: The threshold for significance is *p* < 0.05.

^a^Represent the use of two independent sample *t*‐test.

^b^Represents the use of chi‐square test.

^c^Represents the use of Mann–Whitney *U* test.

### 2.3. Imaging Collection

Using a 3.0T MR imaging system (Signa; GE Healthcare, WI) with an 8‐channel head coil, all patients underwent resting‐state MRI, which included both conventional and advanced sequences. Ax 3D‐BRAVO T1‐weighted imaging scans based on a gradient echo sequence with imaging parameters set as follows: slice thickness = 1.2 mm, interslice distance = 0 mm, TR/TE = 7.8/3 ms, FOV = 240 × 240 mm, flip angle = 15°, 256 × 256 matrix, and number of slices = 248. DTI data were acquired using a single‐shot spin‐echo planar imaging sequence with the following parameters: TR = 8000 ms, TE = 99.3 ms, NEX = 1, slice thickness = 4 mm, interslice distance = 0 mm, 128 × 128 matrix, FOV = 240 × 240 mm, diffusion tensor 16 directions, minimum *b*‐value = 0 s/mm^2^, and maximum *b*‐value = 1000 s/mm^2^.

### 2.4. Imaging Processing

#### 2.4.1. DTI Data Processing and Network Construction

PANDA software developed by the School of Brain and Cognitive Science, Beijing Normal University [22] was used for DTI data preprocessing and white matter network construction. Preprocessing steps involved brain extraction, eddy current, and motion correction. Each subject underwent whole‐brain deterministic tractography using the FACT algorithm in a natural diffusion space. Regions were defined by tracing voxels with FA > 0.2, terminating tractography at angles >35° or FA < 0.2. Group A utilized a 2‐year‐old infant atlas from UNC for node identification [25, 31]. In group B, we used ALL atlas to divide the entire brain into 90 regions and defined nodes in the white matter. For each subject, structural images were confocal with b0 images in native diffusion space using linear transformation and then nonlinearly converted to T1 templates in Montreal Neurological Institute (MNI) space. The derived inverse transformation parameters were then reversed and used to distort the AAL atlas from MNI space to local diffusion space, where discrete marker values were retained by the nearest neighbor interpolation method. A single b0 image and transformed AAL atlas were observed in a single space to ensure that there were no significant mismatch errors [23, 26–28]. The registration effect of all data were repeatedly confirmed by three investigators with rich experience in data processing in order to ensure the reliability of the results. To identify the edges of the white matter network, we identified structural binding if at least three fiber bundles were located between two brain regions. Finally, we obtained an unweighted binary network represented by a symmetric anatomical 90 × 90 matrix for each participant.

#### 2.4.2. Global and Nodal Topological Properties

GRETNA software (http://www.nitrc.org/projects/gretna/) was used for network analysis, with global network properties and regional network properties as critical parameters.

Uses and interpretations of network measures, as well as the specific calculation formulas, refer to relevant reviews [32] and literature [33]. We adopted a single fixed‐threshold strategy, binarizing the original fiber number (FN) matrix using a threshold of three [34, 35]. Specifically, for any pair of brain regions, if the number of streamlines connecting them was greater than or equal to three, the connection was retained (assigned a value of one); if the number was less than three, the connection was removed (assigned a value of zero). This threshold was chosen to exclude spurious connections while preserving sufficient anatomical connectivity to form a biologically meaningful network. The binarized matrices were then imported into the GRETNA software, where we computed a series of global and local graph‐theoretical metrics, including global efficiency (Eglob), local efficiency (Eloc), clustering coefficient (Cp), and characteristic path length (Lp). Global network properties include small‐world index (Cp, characteristic Lp, standardized Cp *γ*, standardized characteristic path length *λ*, and small‐world degree *σ*) and network efficiency (Eglob and Eloc). The small‐world index assesses global information integration efficiency and local information transmission efficiency among nodes. Cp measures network clustering, while *σ* > 1 signifies small‐world characteristics. *λ* represents global integration, *γ* indicates information shunting ability, and Lp is the average shortest Lp between all node pairs. Eglob denotes information transmission across distant brain regions [36], while Eloc reveals the degree of fault tolerance of the network.

Nodal properties include betweenness centrality (BC), degree centrality (DC), and nodal efficiency (Ne) (global and local). BC measures a node’s role in communication by counting shortest paths passing through it. Higher BC signifies greater communication importance [32]. DC indicates node connection intensity and influence in the network. Higher values imply greater impact on information flow. Node efficiency reflects information transmission efficiency among network nodes.

#### 2.4.3. Subnetwork Analysis

Large‐scale brain structural connectivity can be modeled as networks or graphs [37]. To identify significant group differences in this architecture, we employed the network‐based statistic (NBS) approach. Specifically, the FN matrices were analyzed using NBS with a two‐sample *t*‐test as the statistical basis at each connection, and nonparametric permutation tests (threshold = 2.6, *p* < 0.01, 10,000 permutations) were further applied to detect significant group differences in connectivity.

### 2.5. Statistical Analysis

Demographic comparisons used SPSS v26.0. Global properties were analyzed with *t*‐test/Mann–Whitney *U* test for normal/nonnormal data. Nodal properties were assessed using independent sample *t*‐tests and FDR in GRETNA for multiple comparisons. Significance threshold: *p* = 0.05.

## 3. Results

### 3.1. Global Topology Parameters

**Figure 1:**
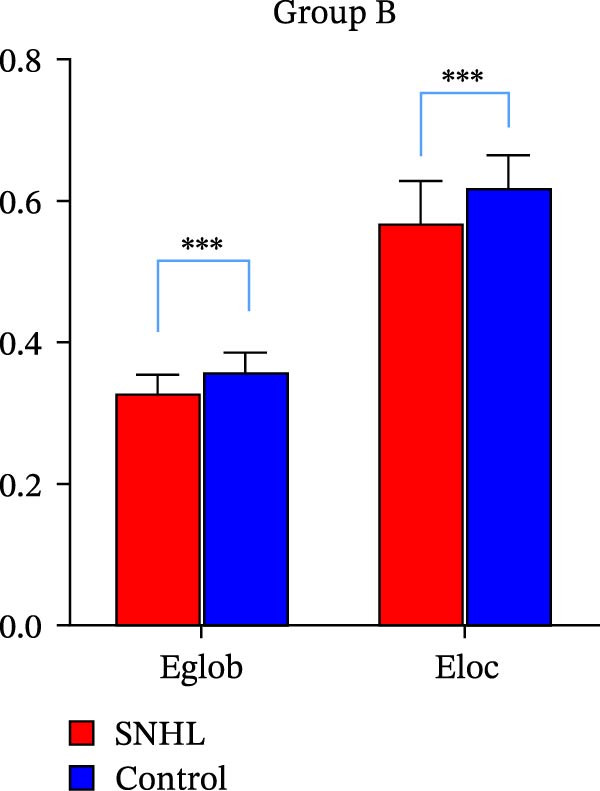
Network efficiency parameters in group B.  ^∗∗∗^Indicates statistical difference, with *p* < 0.001.

**Figure 2:**
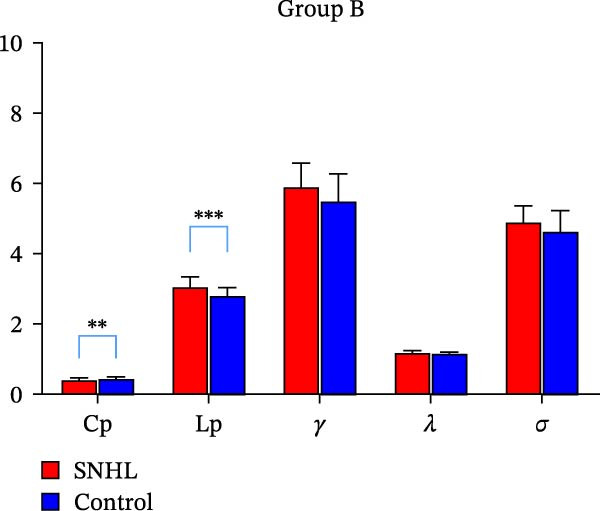
Differences in small‐world parameters in group B.  ^∗∗^Indicates statistical difference, with *p* < 0.01 and  ^∗∗∗^indicates statistical difference, with *p* < 0.001.

In children with SNHL from Group A, no statistically significant differences in global topology parameters were observed compared to the normal control group (Table 2). In contrast, Group B children with SNHL demonstrated reductions in the network efficiency parameters—Eglob and Eloc (Figure 1). They also showed decreases in certain small‐world parameters, such as the Cp, along with an increased characteristic Lp relative to the control group (Figure 2). We observed that the small‐worldness sigma (*σ*) was greater than one in all participants, indicating that brain networks in both the SNHL and normal control groups exhibit a small‐world architecture.

**Table 2:** Global topology parameters.

Global network parameters	Group A				Group B			
	SNHL	Control	*p*	*t*/*U*	SNHL	Control	*p*	*t*/*U*
Cp	0.40 ± 0.063	0.41 ± 0.053	0.891	884.000	0.43 ± 0.041	0.46 ± 0.032	**0.002** ^∗∗^	3.265
Lp	3.12 ± 0.552	2.96 ± 0.373	0.296	778.000	3.07 ± 0.276	2.82 ± 0.221	**<0.001** ^∗∗∗^	−4.053
*γ*	4.88 ± 0.764	4.73 ± 0.776	0.374	0.896	5.92 ± 0.660	5.51 ± 0.764	0.090	398.000
*λ*	1.17 ± 0.044	1.17 ± 0.049	0.878	0.154	1.20 ± 0.046	1.18 ± 0.028	0.085	396.000
*σ*	4.15 ± 0.554	4.02 ± 0.535	0.292	0.652	4.91 ± 0.452	4.65 ± 0.575	0.164	421.000
Eglob	0.33 ± 0.053	0.34 ± 0.043	0.232	0.135	0.33 ± 0.028	0.36 ± 0.029	**<0.001** ^∗∗∗^	4.101
Eloc	0.53 ± 0.098	0.55 ± 0.089	0.402	802.000	0.57 ± 0.062	0.62 ± 0.048	**<0.001** ^∗∗∗^	3.610

*Note*: The bold values indicate statistically significant results.

^∗^Indicates statistical difference, with *p* < 0.05.

^∗∗^Indicates statistical difference, with *p* < 0.01

^∗∗∗^Indicates statistical difference, with *p* < 0.001.

### 3.2. Nodal Topology Parameters

The results of node topology parameters show that no brain regions with enhanced nodal metrics were identified in Group A compared to the normal control group. Children with SNHL showed reductions in nodal BC in the left supplementary motor area (SMA.L) and in nodal local efficiency (Nle) in the left middle temporal gyrus (MTG.L) relative to the normal controls (Figure 3).

Figure 3Differences in node‐level topology between group A and healthy control in children with SNHL. (a) Brain regions with differences in betweenness centrality (BC). (b) Brain regions with differences in nodal local efficiency (Nle). Blue nodes: decreased nodal metrics.

**Figure:**
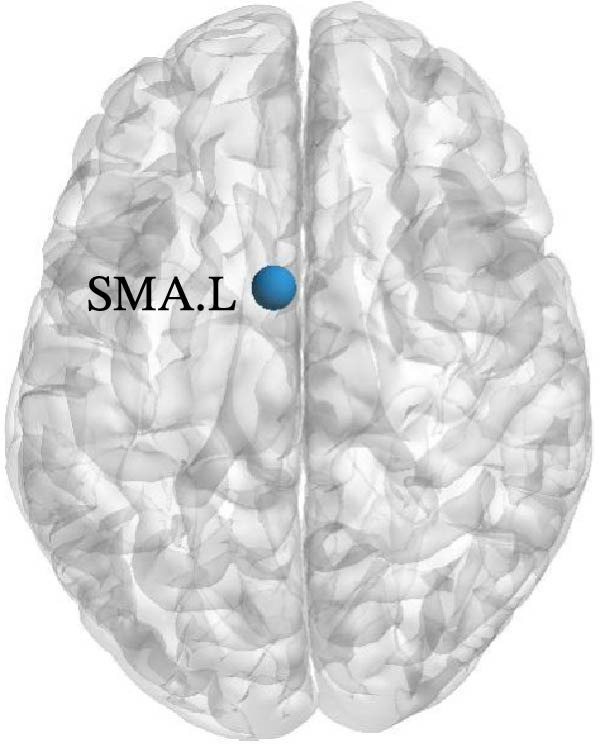
(a)

**Figure:**
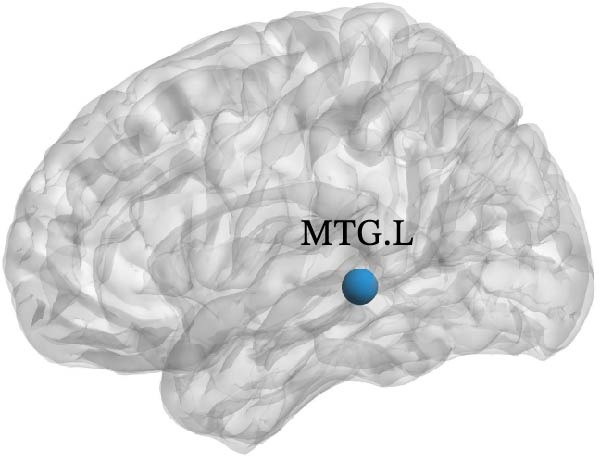
(b)

In Group B, children with SNHL exhibited the following changes compared to the normal control group: (1) Decreased DC in the left medial superior frontal gyrus (SFGmed.L), left inferior temporal gyrus (ITG.L), and right dorsolateral superior frontal gyrus (SFGdor.R); (2) Reduced Ne in the SFGdor.R, right putamen (PUT.R), right precentral gyrus (PreCG.R), right MTG (MTG.R), right SMA (SMA.R), as well as in the SFGmed.L, left SFGdor (SFGdor.L), left middle frontal gyrus (MFG.L), SMA.L, left paracentral lobule (PCL.L), and left calcarine cortex (CAL.L); (3) Decreased Nle in the right ITG (ITG.R); (4) Increased nodal shortest Lp (NLp) in the SFGdor.R, SMA.R, PreCG.R, right postcentral gyrus (PoCG.R), and MTG.R, along with the SFGmed.L, SFGdor.L, MFG.L, SMA.L, PCL.L, and CAL.L (Figure 4). Notably, the brain regions showing increased NLp largely overlapped with those exhibiting decreased Ne. For more details on the nodes with significant differences (e.g., coordinate information), please refer to Table S1.

Figure 4Differences in node‐level topology between group B and healthy control in children with SNHL. (a) Brain regions with differences in degree centrality (DC). (b) Brain regions with differences in nodal efficiency (Ne). (c) Brain regions with differences in nodal local efficiency (Nle). (d) Brain regions with differences in nodal shortest path length (NLp). Blue nodes: decreased nodal metrics. Red nodes: increased nodal metrics.

**Figure:**
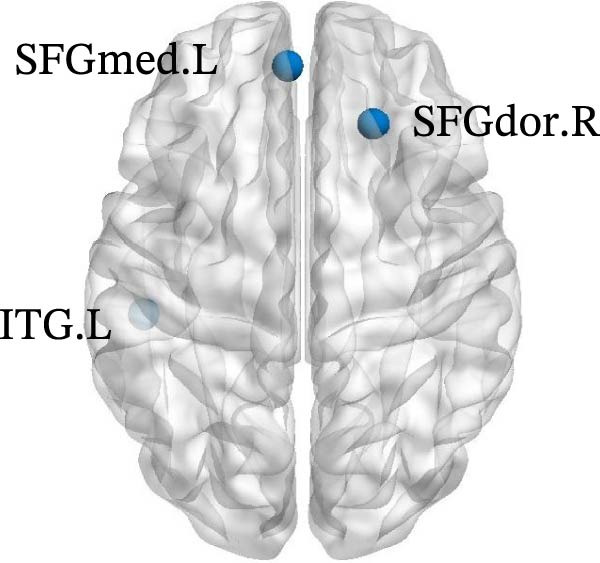
(a)

**Figure:**
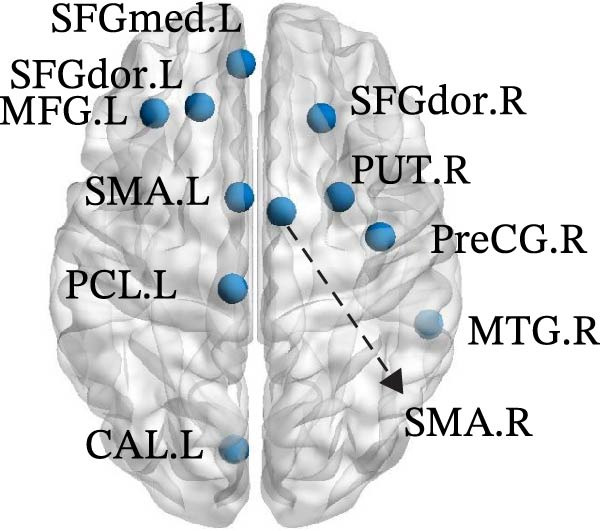
(b)

**Figure:**
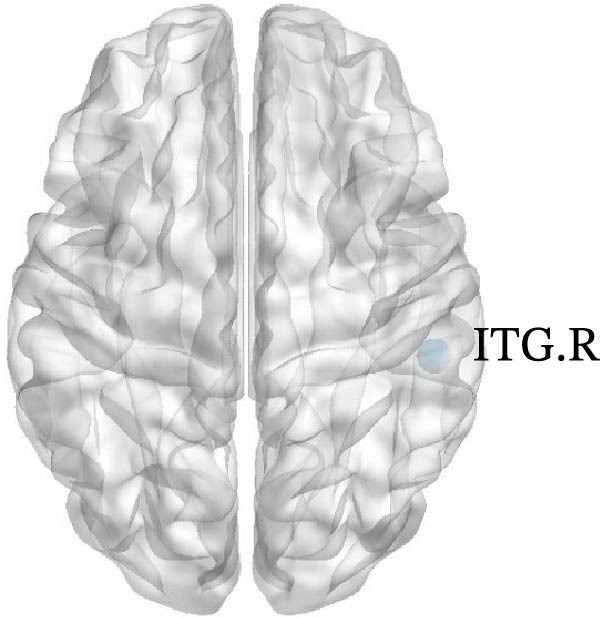
(c)

**Figure:**
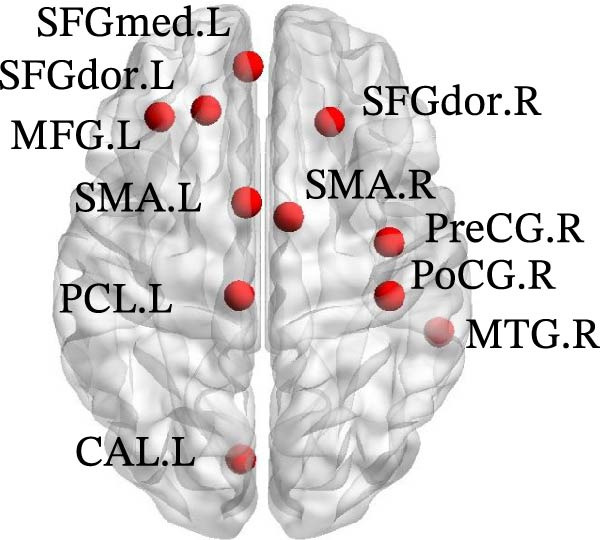
(d)

### 3.3. Structural Connectivity

**Figure 5:**
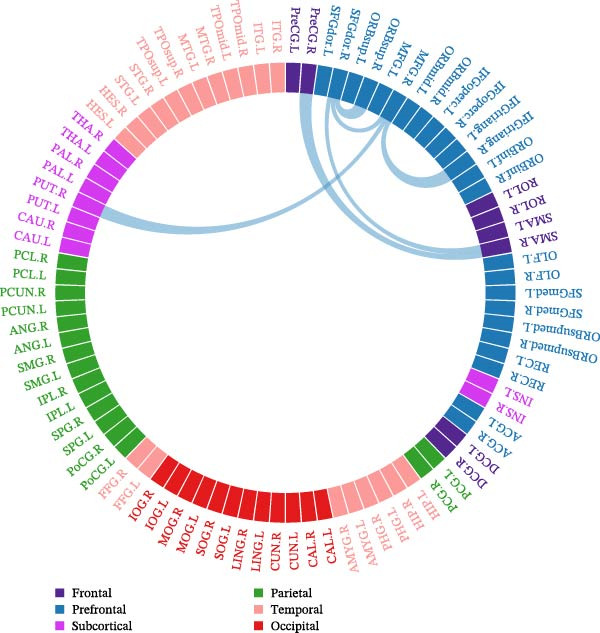
Evaluation of edge connectivity differences using NBS.

No abnormal subnetwork connections were observed in group A. No enhanced connectivity was found in group B compared with the control group, but decreased connectivity in brain regions was found, which was indicated by blue lines. In group B, SNHL children had a neural network with reduced connection strength composed of six nodes and six edges (Figure 5).

Nodes included SFGdor.L, SFGdor.R, left posterior cingulate gyrus (PCG.L), MFG.L, SFGmed.L, and right SFGmed (SFGmed.R) (Figure 5).

## 4. Discussion

In this experiment, we set 4 years as the cutoff age and divided children into a within auditory sensitive period group (group A) and a beyond auditory sensitivity period group (group B), then compared SNHL children with normal children for changes in white matter network. Our first main findings were that compared with age‐matched normal children, SNHL children in group B showed extensive white matter network changes, while no differences were found in small‐world index, network efficiency, or structural connectivity in group A. The above results confirmed our hypothesis of white matter network impairment in deaf children beyond the auditory sensitive period. Another finding was that brain regions with reduced nodal properties in group B were also brain regions with reduced connectivity in the subnetwork analysis, indicating that most of the brain regions with abnormalities in this study were associated with higher‐order cognitive function.

### 4.1. Global Topology Parameters

Consistent with Cui et al. [30], SNHL children in group A did not show changes in global topology parameters, indicating that the structural network of SNHL children within the auditory sensitive period maintains a greater potential for information transmission. Another study also demonstrated that CI during this period tended to benefit more than after this period [38].

Compared with children with normal hearing, SNHL children in group B appeared to have a wide range of Eglob reductions and small‐world attribute changes. The human brain is considered a complex small‐world network that exhibits a balance between information integration (*λ*, Lp, and Eglob) and information separation (*γ*, Cp, and Eloc). Normal small‐world networks possess high Eglob and Eloc [23, 39, 40]. White matter networks in Group B SNHL patients exhibited decreased Cp and increased Lp. The reduction in Cp indicates impaired modularity of local connectivity in the relevant brain regions. The absence of auditory stimulation leads to weakened internal connection strength and reduced connection density of the subnetworks (such as auditory‐related networks and cognitive control networks) that should have been closely coordinated. Lp is a characteristic of global network connectivity; the elevated Lp may be attributed to the degeneration of neural fiber tracts responsible for information transmission. Meanwhile, low Eglob suggests reduced efficiency in long‐range brain connections in SNHL patients. The increase in Lp and decrease in Eglob reflect diminished efficiency in information transmission and interaction between brain regions, ultimately resulting in impaired information processing [23, 41, 42].

### 4.2. Nodal Topology Parameters

Nodal topology parameters, such as DC, Ne, and NLp, reflect the importance and influence of nodes within a brain network. DC measures the connectivity of a node, Ne assesses its information transfer capacity, and a shorter NLp indicates greater efficiency of information flow [43]. Our study found significant abnormalities in nodal topology parameters between children with SNHL and normal‐hearing children across multiple core brain regions. These parameters include DC, Ne, Nle, and NLp. Such abnormalities were especially pronounced in SNHL children who were beyond the auditory sensitivity period. Moreover, the aberrant nodal regions were primarily concentrated in areas associated with the frontal and temporal lobes.

The SFGmed, SFGdor, and MFG are all part of the prefrontal cortex, which has been shown to support a range of cognitive functions such as executive control, emotion regulation, attention, and decision‐making [44, 45]. The SMA and PCL are also key components of the frontal functional network, responsible for speech motor planning, visuospatial working memory, complex motor planning and coordination, speech production, somatosensory‐motor integration, and emotional processing [46, 47]. In this study, decreased DC and Ne along with increased NLp were observed in the SFGmed.L. The SFGdor.R showed reduced DC, while bilateral SFGdor exhibited decreased Ne and increased NLp. Reduced Ne and elevated NLp were also found in the MFG.L, PCL, and bilateral SMA. These findings suggest a weakened hub role and decreased information transfer efficiency of these nodes within the whole‐brain network, reflecting impaired coordination within the frontal subnetwork. Moreover, most of these brain regions are located within the association cortex, which supports uniquely human cognitive, socioemotional, and mentalizing abilities. The declining trends in their nodal metrics may increase the risk of delayed cognitive development and emotional regulation difficulties in children with SNHL, contributing to individual variability in executive and psychosocial functioning [2, 48].

Abnormalities in the temporal lobe network also reflect changes in brain regions following auditory deprivation. The ITG plays a crucial role in higher‐order functions such as visual object perception and recognition, lexical and phonological decision‐making, and the ventral visual pathway [49–51]. The MTG, adjacent to Wernicke’s area, is involved in speech comprehension, auditory memory, and emotional sound processing, and is responsible for semantic parsing of complex sentences and short‐term storage of auditory information [52, 53]. Deficits in the ITG and MTG may lead to reduced fluency in multisensory information processing, impaired multimodal sensory integration, and dysfunction in complex visual perception. In this study, decreased DC was observed in the ITG.L, reduced Ne in the ITG.R, and decreased Ne along with increased NLp in the right MTG. These findings suggest that auditory deprivation results in decreased information transfer efficiency in the ITG and MTG, which may underlie the delayed speech comprehension and difficulties in emotional sound recognition observed in children with SNHL.

### 4.3. Structural Connectivity

In subnetwork analysis, no differences in structural connectivity were observed in group A, pointing out the absence of significant white matter network connectivity abnormalities among children within the auditory sensitivity period. On the other hand, a neural circuit with decreased connection strength was found in group B, consisting of seven brain regions and six edges.

Among the six brain regions with altered connection strength in Group B, four also exhibited abnormalities in nodal parameters: the SFGdor.R, PreCG.R, SMA.R, and PUT.R. The remaining three brain regions showed no changes in nodal attributes. This indicates that, although the nodal properties of these regions remained unchanged, their connectivity with other brain regions became abnormal. We attribute this to long‐term hearing loss [54, 55].

Notably, decreased connections were primarily concentrated in the frontal lobe, especially the prefrontal cortex. The SFGdor.R and MFG.R showed the highest number of affected connections, each exhibiting weakened connectivity with three other brain regions. We propose that these two regions serve as key nodes within this subnetwork. The SFGdor and MFG are critical components and hubs of the central executive network (CEN), which is responsible for goal‐oriented integration of multimodal information, regulation of higher cognitive processes, and coordination of goal‐directed behavior and emotional responses [56–58]. When the brain undergoes pathophysiological changes, the dense structural connections of these hubs become particularly vulnerable. Weakening of the structural connections in these key hubs disrupts the structural framework of the entire cognitive control and emotional regulation system, leading to a collapse in network organization efficiency—manifested as abnormalities in nodal topology parameters. A resting‐state fMRI study of prelingually deaf adults found that not only was functional connectivity within the CEN reduced, but there were also abnormal interactions with the default mode network and salience network. These alterations ultimately contribute to impairments in executive function and emotional regulation [59–61]. Moreover, nodes such as SFGdor.R, SMA.R, and PUT.R belong to the abnormal “prefrontal‐striatal” circuit. We propose that the reduced connection strength within this subnetwork may be one of the mechanisms underlying higher‐order functional impairments in infants with SNHL due to auditory deprivation [55, 62, 63].

Furthermore, this study found that the majority of brain regions exhibiting reduced connectivity are located in the prefrontal cortex. The prefrontal cortex does not function in isolation; rather, it operates through looped circuits connecting with other brain regions to support integrated functions, which significantly influence psychological and behavioral outcomes. Previous studies have reported a higher incidence of psychiatric conditions in children with SNHL, such as autism spectrum disorder, behavioral problems, and attention deficits [54]. A longitudinal study also suggested a significant association between childhood hearing loss and reduced well‐being and self‐esteem, as well as increased anxiety and depression. Therefore, we hypothesize that abnormalities in prefrontal neural circuits may be one of the contributing factors to atypical psychological development in children with SNHL [62, 64].

### 4.4. Changes in Topological Properties and Potential Cognitive Loss After Auditory Sensitive Period

Our study proposed that SNHL children within the auditory sensitivity period showed decreased nodal attributes in hearing and cognitive‐related brain regions, but without global attributes and decreased subnetwork structures, indicating that white matter network structures between brain regions had not yet shown significant changes at the whole brain level, which was explained as an optimistic potential for information transmission by some literature [30]. SNHL children beyond the auditory sensitivity period tend to have more pronounced and extensive white matter network changes. Our study reveals the outcomes of neuroplastic remodeling in the brains of children with SNHL as they adapt to a sound‐deprived environment—a process that entails potential costs. Its development follows a progression: starting with a latent decline in Ne during the auditory sensitive period (Group A) to the disruption of key structural connections and the degeneration of global network properties in brains beyond that sensitive period (Group B). This is not a static condition but a dynamically evolving process that extends with the duration of auditory deprivation, likely representing a gradual shift from functional compensation to structural decompensation. We characterize the SNHL‐related brain features emerging from this process as the “Deaf Brain Features.”

In addition to detecting decreased nodal topological attributes in auditory‐related brain regions, this study also found that most brain regions in group B with decreased connectivity and abnormal nodal attributes were linked with higher‐order cognitive function (e.g., language, attention, memory, social communication, affect, reward, and self‐cognition). These networks have emerged even earlier in the neonate [65]. Approximately 30% of deaf children have additional disabilities, with cognitive disorders as the most common one [66]. Although speech and hearing can be improved through artificial interventions, such as hearing aids and cochlear implants, children with hearing loss still face risks of various cognitive dysfunctions, including working memory deficits and executive function impairments, which can negatively impact their subsequent education and employment [64, 67]. Once the auditory sensitive period is missed, the formation of deaf brain features in children with SNHL becomes more pronounced, exerting a greater impact on higher‐order cognitive regions. Therefore, hearing stimulation should be introduced as early as possible before the auditory sensitive period to reduce extensive white matter network damage. Our study provides imaging evidence for the existence of this critical developmental window and further clarifies the necessity of early intervention for SNHL.

Numerous previous studies on CI outcomes support the conclusion that SNHL should be intervened as early as possible. Research by Culbertson et al. [68] indicates that younger age at CI is associated with stronger auditory development over time. Early implantation not only benefits auditory development but also promotes the development of advanced cognitive functions such as language. Dettman et al. [69] found that early CI provides favorable conditions for language learning and communication skills in children with SNHL. However, existing studies have not yet reached a consensus on the optimal timing for CI. Based on our findings, we hypothesize that this “timing‐dependent” effect of the intervention may stem from the degree to which deaf brain features have developed: during the sensitive period, the deaf brain features remain in a compensable state, where restoring auditory input via CI alone can leverage brain plasticity to repair abnormalities in auditory‐related nodes; after the sensitive period, the deaf brain features shift to a decompensated state, necessitating not only the repair of the auditory pathway but also targeted rehabilitation strategies to address structural damage in subnetwork circuits.

To some extent, the results of our study provide more precise neuroimaging evidence for determining the timing of CI. However, the specific neural mechanisms still require validation and supplementation through multidimensional research. Future clinical practice should move beyond the singular goal of “restoring hearing” toward a new paradigm of “remodeling brain networks.” This includes utilizing preoperative brain network imaging for outcome prediction and integrating interventions targeting advanced cognitive functions into postoperative rehabilitation, thereby maximizing the recovery and activation of neurodevelopmental potential in every child with SNHL.

### 4.5. Limitations and Expectation

Refine study design for better child recruitment, age group definition, and optimal CI age determination. Criteria development for data processing, larger sample sizes for SNHL research consistency, and long‐term follow‐up for comprehensive clinical data are needed.

## 5. Conclusion

By constructing the white matter structural brain network in a large sample of SNHL children, this study identified that SNHL children beyond the auditory sensitive period had abnormally aggravated white matter networks; that is, the formation of deaf brain features was more obvious in SNHL children older than 4 years. However, SNHL children had not undergone extensive changes in global topology parameters and subnetworks in the early phase of the auditory sensitive period. Therefore, hearing stimulation should be introduced to SNHL children as early as possible in order to avoid malformed brain remodeling.

## Author Contributions

**Wenbin Zheng**: conceptualization, validation, manuscript editing and review. **Jiayan Zhuang**: data acquisition and curation, data analysis, methodology, statistical analysis and writing – original draft. **Jin Wang**: data acquisition and curation, data analysis, statistical analysis and writing – review and editing. **Gengbiao Zhang**: data acquisition and curation, software, data analysis, methodology and writing – review and editing. **Hongyi Zheng**: validation, manuscript editing and review. **Lingmei Kong**: data acquisition, manuscript editing and review. **Lexing Huang**: data acquisition, validation, manuscript editing and review. **Juyue Hong**: data acquisition and curation.

## Funding

This work was supported by a grant from the Guangdong Provincial Natural Science Fund (Grant 2024A1515011698), the Joint Research Fund for Enterprise and Basic and Applied Basic Research Programs of Guangdong Province (Grant 2021A1515220112), the Provincial Science and Technology Innovation Strategy Special Project Funding Program (Grant STKJ2023042), and the National Natural Science Foundation of China (Grant 81571627).

## Disclosure

All authors contributed to the article and approved the submitted version. Following the linguistic polishing by AI tools, all authors carefully reviewed the revised content. Additionally, a native English speaker was commissioned to participate in the review process to ensure the accuracy of sentence meanings and the completeness and standardization of linguistic structures. This manuscript has been posted as a preprint on SSRN (https://papers.ssrn.com/sol3/papers.cfm?abstract_id = 4954891) [70].

## Ethics Statement

The study was conducted in accordance with the Declaration of Helsinki and approved by the Ethics Committee of the Second Affiliated Hospital of Shantou University Medical College (Protocol Code 2022‐11, March 11, 2022). Prior to the study, the parents or guardians of all participants were briefed on the MRI safety procedures and experimental protocol and provided their written informed consent.

## Conflicts of Interest

The authors declare no conflicts of interest.

## Supporting Information

Additional supporting information can be found online in the Supporting Information section.

## Supporting information


**Supporting Information** Table S1: Information of brain regions with differences in nodal topological parameters in Group B.

## Data Availability

The data that support the findings of this study are available upon request from the corresponding author.
