# Supplementary material for: Alterations of White Matter Structural Brain Network in Children With Sensorineural Hearing Loss: A Graph Theory Analysis for Auditory Sensitivity Period
Source: Neural Plast. 2026 Mar 13;2026:4263849. doi: 10.1155/np/4263849 (PMC13140368; doi:10.1155/np/4263849)
Supplement: Supplementary file 1 — Supporting Information Table S1: Information of brain regions with differences in nodal topological parameters in Group B. [file NP-2026-4263849-s001.docx]

**Table S1**. Information of brain regions with differences in nodal topological parameters in Group B

| **Group** | **Nodal Topological Parameters** | **Regions** | **MNI coordinates** | | | **P value** |
| --- | --- | --- | --- | --- | --- | --- |
|  |  |  | X | Y | Z |  |
| B | Degree Centrality | SFGdor.R | 21.90 | 31.12 | 43.82 | 0.000261 |
|  |  | SFGmed.L | -4.80 | 49.17 | 30.89 | 0.000297 |
|  |  | ITG.L | -49.77 | -28.05 | -23.17 | 0.000230 |
|  | Nodal Efficiency | PreCG.R | 41.37 | -8.21 | 52.09 | 0.000217 |
|  |  | SFGdor.L | -18.45 | 34.81 | 42.20 | 0.000002 |
|  |  | SFGdor.R | 21.90 | 31.12 | 43.82 | 0.000009 |
|  |  | MFG.L | -33.43 | 32.73 | 35.46 | 0.000153 |
|  |  | SMA.L | -5.32 | 4.85 | 61.38 | 0.000014 |
|  |  | SMA.R | 8.62 | 0.17 | 61.85 | 0.000096 |
|  |  | SFGmed.L | -4.80 | 49.17 | 30.89 | 0.000025 |
|  |  | CAL.L | -7.14 | -78.67 | 6.44 | 0.000042 |
|  |  | PCL.L | -7.63 | -25.36 | 70.07 | 0.000160 |
|  |  | PUT.R | 27.78 | 4.91 | 2.46 | 0.000507 |
|  |  | MTG.R | 57.47 | -37.23 | -1.47 | 0.000061 |
|  | Nodal Local Efficiency | ITG.R | 53.69 | -31.07 | -22.32 | 0.000137 |
|  | Nodal Shortest Path Length | PreCG.R | 41.37 | -8.21 | 52.09 | 0.000212 |
|  |  | SFGdor.L | -18.45 | 34.81 | 42.20 | 0.000008 |
|  |  | SFGdor.R | 21.90 | 31.12 | 43.82 | 0.000031 |
|  |  | MFG.L | -33.43 | 32.73 | 35.46 | 0.000261 |
|  |  | SMA.L | -5.32 | 4.85 | 61.38 | 0.000010 |
|  |  | SMA.R | 8.62 | 0.17 | 61.85 | 0.000042 |
|  |  | SFGmed.L | -4.80 | 49.17 | 30.89 | 0.000046 |
|  |  | CAL.L | -7.14 | -78.67 | 6.44 | 0.000103 |
|  |  | PoCG.R | 41.43 | -25.49 | 52.55 | 0.000499 |
|  |  | PCL.L | -7.63 | -25.36 | 70.07 | 0.000284 |
|  |  | MTG.R | 57.47 | -37.23 | -1.47 | 0.000078 |
| Note: A Bonferroni-corrected threshold of P<0.000556(0.05/90 nodes) was applied for multiple comparisons. | | | | | | |
